# Ground Reaction Forces, Asymmetries and Performance of Change of Direction Tasks in Youth Elite Female Basketball Players

**DOI:** 10.3390/sports12010021

**Published:** 2024-01-08

**Authors:** Jordi Arboix-Alió, Bernat Buscà, Adrià Miró, Chris Bishop, Azahara Fort-Vanmeerhaeghe

**Affiliations:** 1 Department of Sports Science, Ramon Llull University, 08022 Barcelona, Spain; bernatbs@blanquerna.url.edu (B.B.); adriama@blanquerna.url.edu (A.M.); azaharafv@blanquerna.url.edu (A.F.-V.); 2 School of Health Sciences, Ramon Llull University, 08022 Barcelona, Spain; 3 F.C. Barcelona, Sport Performance Area, 08970 Barcelona, Spain; 4 Faculty of Science and Technology, London Sport Institute, Middlesex University, London NW4 4BT, UK; C.Bishop@mdx.ac.uk; 5 Segle XXI Female Basketball Team, Catalan Federation of Basketball, 08950 Esplugues de Llobregat, Spain

**Keywords:** imbalances, ground reaction force, ground contact time, symmetry, agility

## Abstract

The magnitude and direction of inter-limb asymmetries in a change of direction (COD) have increased interest in scientific research in recent years. This present study aimed to investigate the magnitude of asymmetries in an elite youth female basketball sample (n = 18, age = 17.79 ± 0.67 y) and determine its directionality using force platform technology. Participants performed 70° and 180° COD tests analyzing the following variables: time, ground contact time (GCT) and ground reaction forces (GRF) along the anterior–posterior, mediolateral, and vertical axes. Inter-limb asymmetries were evident in both COD tests, with substantial differences observed between limbs (*p* < 0.01). The asymmetry values ranged from 3.02% to 24.31% in COD 180° and from 1.99% to 21.70% in COD 70°, with anterior–posterior GRF consistently exhibiting the highest asymmetry magnitude. Additionally, the directionality exhibited variability between the tests, indicating poor agreement and suggesting the independent directionality of asymmetries across tasks. Moreover, players required more time to complete the COD 180°, the GCT was noticeably longer for the COD 180° than for the COD 70°, and GRF varied across the axis, suggesting that players adapt uniquely to the specific demands of each task. The utilization of force platforms presents a comprehensive approach to assess asymmetries and COD variables performance variables which are “angle-dependent”, which could have important implications for COD screening and effective training interventions.

## 1. Introduction

The capacity to swiftly accelerate, reverse direction, or execute rapid changes in movement, followed by quick re-acceleration is a pivotal aspect of many team sports [1]. These sports involve high-intensity unilateral actions like jumping and sudden changes of direction (COD) during critical moments such as scoring opportunities or offensive and defensive maneuvers, which in turn, can significantly influence the outcomes of matches [2]. Consequently, professionals and researchers are continuously in search of more effective methods to enhance and optimize athletes’ acceleration and re-acceleration capabilities.

The demanding nature of high-intensity unilateral movements in team sports frequently leads to the development of asymmetrical neuromuscular adaptations in the lower limbs [3]. While some experts suggest that these asymmetries pose an injury risk [4,5], only a limited number of studies have explored the connection between these factors [6,7,8]. This concern is particularly pertinent for female athletes, as they exhibit a higher prevalence of inter-limb differences in strength, coordination, and postural control compared to their male counterparts [9]. In recent years, there has been a growing focus on investigating inter-limb asymmetries [10,11]. Current literature highlights that these asymmetries vary not only in magnitude, but also in direction across various tasks and skills [3,12]. Further to this, some studies suggest that side-to-side asymmetries in force or power during unilateral jumping are associated with reduced athletic performance [13]. While the relationship between these factors and COD abilities remains unclear [12,14], it stands to reason that in multi-directional sports, achieving equal proficiency when swiftly changing direction from both limbs would be advantageous, given the unpredictable agility demands of these sports [15].

Among these team sports, basketball is an intermittent team sport defined by a range of unilateral, high-intensity movements, including leaps and CODs, each intimately tied to pivotal junctures within the game’s dynamics [16,17]. Basketball players routinely execute rapid decelerations, COD maneuvers, and explosive sprints to establish favorable positions or to swiftly respond to opponents or the ball’s trajectory [18]. Considering the inherent risks associated with these demanding unilateral actions, such as jumping and CODs, injury prevention is an important part of basketball training programs [19]. Consequently, the systematic evaluation of inter-limb asymmetry, particularly during activities like jumping and COD, emerges as an important strategy to reduce injury risk in basketball players and potentially increase players’ performance [11,20].

While previous research has explored asymmetry in COD speed tasks [15,21], the majority of research has only reported commonly used outcome measures such as total time or the COD deficit. However, the use of force platforms during COD assessments enable a greater depth of information by providing a combination of outcome measures and strategy-based data [22,23]. Thus, with this data, it also becomes possible to conduct a more comprehensive and nuanced analysis of asymmetry. Moreover, force platforms may dissect the forces generated by each limb separately, allowing researchers to precisely quantify the forces exerted by the left and right limb during COD tasks [3]. By gaining insights into how each limb contributes to overall COD performance, researchers can pinpoint specific factors that might be driving why the asymmetry is present in the first instance. Therefore, the primary objective of this study was to improve the current understanding by conducting a comprehensive evaluation of the magnitude and direction of asymmetry in various COD indicators using force platforms technology in a sample of youth elite female basketball players.

## 2. Materials and Methods

A cross-sectional design was used to assess the amount of asymmetry and its direction in different COD variables. Thus, 70° and 180° COD tests with both limbs were conducted at the end of the competitive basketball season (2021–2022).

### 2.1. Participants

Eighteen elite female youth basketball players voluntarily participated in this study, with the following characteristics: age (mean ± standard deviation) of 17.79 ± 0.67 years, body mass of 71.10 ± 7.43 kg, height of 1.82 ± 0.07 m, body mass index of 23.01 ± 1.69 kg·(m^2^)^−1^, sports experience spanning 6.42 ± 1.41 years, and years’ post-peak height velocity at 4.89 ± 0.68 years. To assess biological maturation in a non-invasive manner, Mirwald et al. [24] employed a regression equation utilizing age, body mass, standing height, and sitting height. At the time of the study, all participants were actively enrolled in a four-year talent development program, which typically encompassed 7–9 weekly training sessions and weekend games. Athletes were excluded from participation if they had any injury (acute or chronic) or illness at the time of the tests that prevented them from exerting maximum effort. Before initiating the study, participants and their parents or guardians were provided with comprehensive verbal and written explanations detailing the potential testing-related risks and discomforts. Subsequently, written informed consent was diligently obtained from all participants as well as their respective parents or guardians. This research received ethical approval from the Ramon Llull University Ethics Committee under reference number 1718007D and strictly adhered to the principles outlined in the Declaration of Helsinki (2013 revision, Fortaleza, Brazil).

### 2.2. Design and Procedures

One week prior to data collection, all participants received comprehensive familiarization with the test protocols and procedures. During the days of testing, a standardized warm-up was employed for ensuring the quality of the assessments. These warm-up drills started with a 5 min session of cardiovascular exercise aimed at achieving a rate of perceived exertion (RPE) between 5 and 6. Subsequently, participants engaged in a 6 min period of multidirectional displacements, followed by 4 min of dynamic stretching exercises, including activities like walking lunges and high knee lifts while taking side steps. Additionally, the warm-up included 3 min of progressively intensifying displacements that involved various movements such as changes of direction, jumps, and acceleration/deceleration exercises. Throughout the warm-up, the participants were under the vigilant supervision of a certified strength and conditioning coach, who ensured proper technique and offered consistent feedback. To acquaint the participants with the test procedures, they were granted 3 practice trials, each conducted at perceived effort levels of 70%, 85%, and 100% of their maximal capacity. A rest interval of 2 min was set between the final practice test and the start of the first official test. To eliminate potential biases, both the test order and the sequence of participants were randomized using a “true random number generator” program.

#### 70° and 180° COD Tests

**Figure 1:**
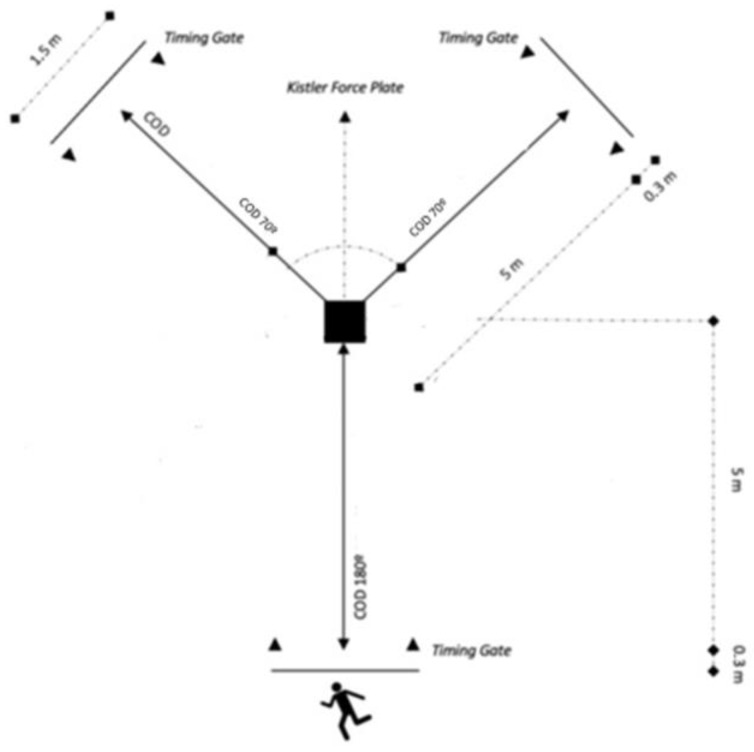
Schematic representation of the change direction tests.

For the assessment of COD capabilities, participants were instructed to execute a sprint for five meters, followed by a 70° or 180° turn, and culminating with another five-meter sprint (Figure 1). The COD time was recorded using timing gates (Witty, Microgate, Bolzano, Italy). Each COD test was initiated with the participants standing, their preferred foot positioned forward, and starting 0.5 m behind the initial gate [25]. The gates were positioned at intervals of 1.5 m and situated 1.3 m above the ground. Subsequent analysis was conducted on the participants’ fastest trial in each direction.

**Figure 2:**
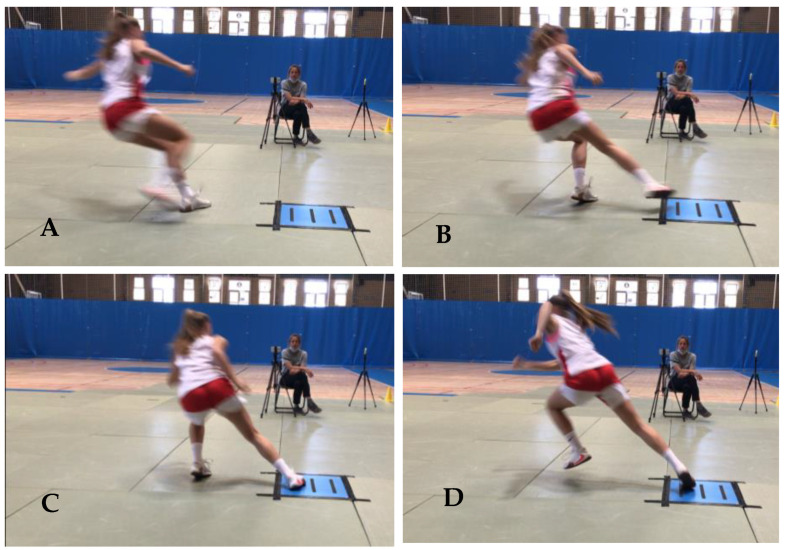
Player performing the COD 180°.

**Figure 3:**
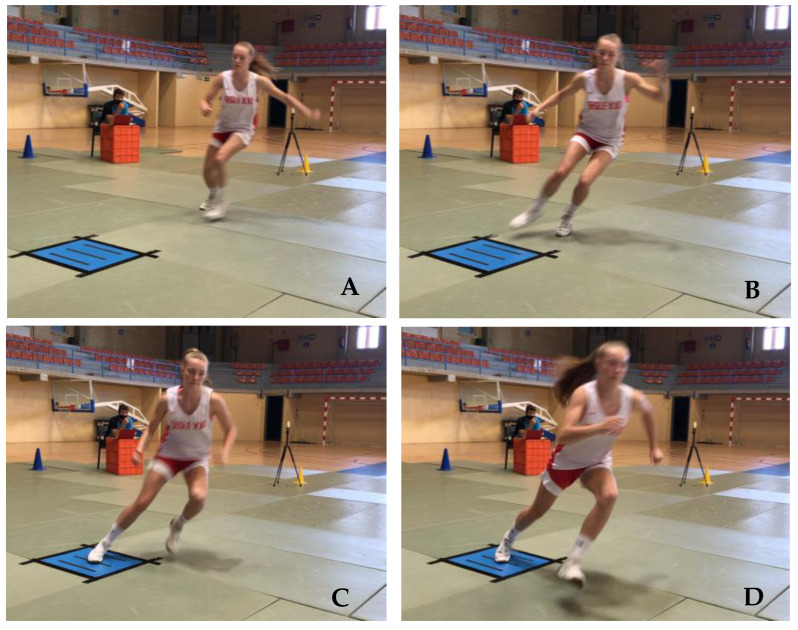
Player performing the COD 70°.

In the pursuit of acquiring ground reaction force data during the COD tests, participants executed the directional change with the push-off foot (located externally) making full contact within the parameters of the force plate (Figure 2 and Figure 3). Trials were excluded from analysis if the participants landed outside the designated area of the force plate. Ground reaction forces (GRF) along the x (anterior–posterior), y (mediolateral), and z (vertical) axes, as well as the ground contact time (GCT), were quantified using a force plate (Kistler 9260AA, Winterthur, Switzerland) equipped with an accompanying data acquisition system (Kistler 5695b, Winterthur, Switzerland).

### 2.3. Statistical Analysis

Statistical analyses were conducted using SPSS (Version 25 for Windows; SPSS Inc., Chicago, IL, USA). Mean values along with their standard deviations (SD) were computed for all variables. The normality of the tested variables was assessed using the Shapiro–Wilk test. Additionally, the within-session reliability of test measures was evaluated using a two-way random intraclass correlation coefficient (ICC) with absolute agreement (95% confidence intervals) and the coefficient of variation (CV). ICC values were categorized as follows: >0.9 = excellent, 0.75–0.9 = good, 0.5–0.75 = moderate, and <0.5 = poor [26]. CV values were deemed acceptable if they were below 10% [27]. The asymmetry index (ASI) was determined using the following formula [4]:

$$\mathrm{ASI}\%=\frac{Highest\;Performing\;Limb-Lowest\;Performing}{Highest\;Performing\;Limb}\times 100$$

The highest performing limb (HPL) was defined as the side with the higher value for each task, while the lowest performing limb (LPL) was defined as the side with the lower. To identify differences between limbs, paired sample *t*-tests were used to compare HPL and LPL. The magnitude of the difference was determined using Cohen’s *d* effect sizes (ES) with 95% confidence intervals [28]. Values were interpreted as <0.20 = trivial; 0.20–0.60 = small; 0.61–1.20 = moderate; 1.21–2.0 = large and >2.0 = very large, following the suggestions from Hopkins et al. [29]. To determine the direction of asymmetry, an “IF function” was incorporated into the formula within the Microsoft Excel software (version 2311): *IF(left < right, 1, −1) [11]. Noting that asymmetries may favor either side depending on which limb scores more, a Kappa coefficient was calculated to determine how consistently asymmetries favored the same side between tests. This approach was selected because the Kappa coefficient quantifies the level of agreement between two methods, accounting for any chance agreement [30]. Interpretation of Kappa values followed the criteria proposed by Viera and Garrett [31], where ≤0 indicated poor agreement, 0.01–0.20 suggested slight agreement, 0.21–0.40 denoted fair agreement, 0.41–0.60 represented moderate agreement, 0.61–0.80 signified substantial agreement and 0.81–0.99 indicated almost perfect agreement.

## 3. Results

Descriptive statistics and reliability measures for all tests are shown in Table 1. Almost the tests showed good within-session ICC values (≥0.9) and had acceptable consistency with CV values < 10%.

When comparing COD variables between both tests, players require more time to complete the COD 180° test in comparison with the COD 70° test (*p* < 0.01; ES = 3.62). Additionally, the GCT were noticeably longer for the COD 180° test compared to the COD 70° test (*p* < 0.01; ES = 2.55 for the right limb and ES = 3.66 for the left limb). In terms of ground reaction forces values, the COD 180° test exhibited higher values for GRFx (*p* < 0.01; ES = 1.04 for the right limb and ES = 1.41 for the left limb), while the COD 70° test displayed higher values for GRFy (*p* < 0.01; ES = 5.62 for the right limb and ES = 4.31 for the left limb), and GRFz (*p* < 0.01; ES = 2.44 for the right limb and ES = 2.07 for the left limb).

Comparisons between the different variables from HPL and the LPL (Table 2 and Table 3) showed significant differences across all variables (*p* < 0.01) with a moderate to large difference magnitudes (ES > 0.82 in COD 180° and ES > 1.31 in COD 70°, respectively. The mean asymmetry values ranged from 3.02 to 24.31 in COD 180° and from 1.99 to 21.70 in COD 70°. In both tests, GRFx variable reported the highest asymmetry value.

**Figure 4:**
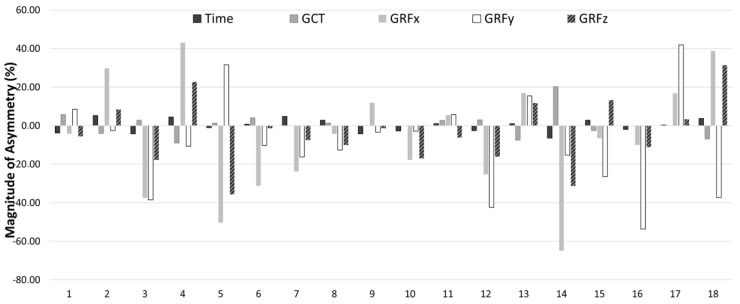
Percentage of asymmetry index (ASI) for each participant and variable (positive = right leg dominance; negative = left leg dominance) for the COD 180°.

**Figure 5:**
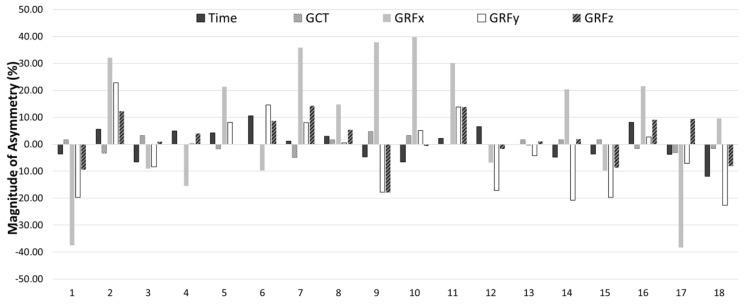
Percentage of asymmetry index (ASI) for each participant and variable (positive = right leg dominance; negative = left leg dominance) for the COD 70°.

Kappa coefficients and the descriptors of how consistently the asymmetry favored the same limb for each COD variable between COD 180° and COD 70° are presented in Table 4. The results showed that asymmetries rarely favored the same side between tests (Kappa = −0.111 to 0.143), showing different directionality depending on the COD angle. Figure 4 and Figure 5 present the individual asymmetries for each test. In these figures, positive values mean right limb predominance, while negative values a left limb superiority.

## 4. Discussion

The present study aimed to determine the magnitude and direction of asymmetries in different CODs considering the temporal performance and the ground reaction forces of the two tests in youth elite female basketball players.

Results revealed significant differences between COD 70° and COD 180° tests in terms of completion time, GCT and GRF. As expected, players required more time to complete the COD 180° than the COD 70°. How can it be otherwise, the GCT was noticeably longer for the COD 180° than for the COD 70°. These findings suggest that larger COD angles demand greater time to execute, potentially due to the increased braking forces and biomechanical demands associated with changing direction over a wider angle. These findings align with Dos’Santos et al. [32], who reported that when the COD angle increased, performance variables declined with reductions in velocity profiles and longer GCTs. In the present study, the differences in GCT between both tests are particularly interesting from a biomechanical standpoint. Longer GCT in COD 180° indicates that players spent a greater proportion of their time in ground contact during this task. This finding could be attributed to the increased braking forces and the need for a more controlled and balanced deceleration and acceleration phase when changing direction by 180°. In contrast, the COD 70° task may allow for a more rapid and explosive change in direction, resulting in a shorter GCT [32]. Similarly, Gonzalo-Skok et al. [33] suggested that larger angles than 90° require more significant reductions in velocity (i.e., braking) as well as greater reacceleration, influencing parameters like GRF or GCT. When examining the GRF data, the present study found that the COD 180° test yielded higher values for the anterior–posterior ground reaction forces (GRFx), while the COD 70° test exhibited higher values for the mediolateral (GRFy) and vertical (GRFz) forces. This discrepancy in force distribution suggests that players rely more on horizontal force production during the COD 180° test, possibly due to the need for rapid total deceleration and re-acceleration in a shorter span [32,34]. Conversely, the COD 70° task places a greater emphasis on vertical and mediolateral forces, probably because there is less needed to brake beforehand, emphasizing the importance of lateral propulsion maintaining the velocity. This variability in ground reaction force distribution across the three axes underscores the “angle-dependent” nature of COD tasks, with demands varying according to the specific type of COD [32]. Another factor potentially influencing the GRF exerted are the differences of the body posture and the center of gravity height achieved by the players during the ground contact phase of the foot responsible of changing the direction. During the testing session, a deepest knee flexion was observed (although not assessed) for the 180° maneuver, thus explaining the longer contact times and the grater GRF on the *x*-axis (antero-posterior) [34]. Nevertheless, the highest GRF on the *z*-axis (vertical), associated with higher running speeds [35], were found for the 70° COD.

The findings of the present study are in agreement with previous research highlighting the presence of inter-limb asymmetry in youth elite female basketball players when performing COD tasks [36,37]. In both the COD 180° and COD 70° tests, significant differences were observed between HPL and LPL across all variables. These differences were moderate to large in magnitude, suggesting that inter-limb differences are a prevalent and substantial characteristic of COD performance in this population. This finding is consistent with previous studies that have reported similar inter-limb differences in force variables during unilateral movements [11]. Interestingly, the asymmetry magnitude of the different COD indicators was similar across both COD tasks. In the COD 180° test, asymmetry values ranged from 3.02 to 24.31, while in the COD 70° test, ranged from 1.99 to 21.70. Notably, the GRFx variable consistently exhibited the highest asymmetry value in both tests. This finding suggests that players may generate significantly different levels of horizontal forces with their HPL and LPL, potentially impacting their ability to execute COD tasks effectively [21]. Indeed, the coordination of braking and reaccelerating phases, mainly expressed as GRFx and GCT, characterize an effective COD.

Finally, this present study identified a lack of consistency in the direction of asymmetry between the COD 180° and COD 70° tests. The Kappa coefficients, which assess the consistency of the direction of asymmetry between tests, indicated poor agreement. Asymmetries rarely favored the same side between both tests, further highlighting the variability in directionality. This variability suggests that athletes may adapt differently depending on the specific demands of the task, resulting in different dominant limbs and varying asymmetry patterns. Moreover, this lack of consistency was also evident when comparing the directionality of the different indicators for the same COD task. Provably, the nature of the sample can explain this lack of consistency. The movement pattern variability of the young players is usually higher than those of the adults [38]. For this reason, a consistent laterality in COD tasks is difficult to find at the sample’s age, although the players spent more than 10 h per week of basketball training sessions. This discrepancy highlights the importance of conducting measurements across a range of neuromuscular skills, rather than depending solely on a single test to measure inter-limb differences. This comprehensive approach is essential for obtaining a complete and individualized understanding of asymmetry patterns in athletes and having a more individual approach to data interpretation [39]. For example, athlete 17 in Figure 4 or athlete 11 in Figure 5, show consistency in limb dominance for all metrics. Such consistent patterns in limb dominance might signify a potential deficit in COD capacity; thus, it is suggested that a more individual strength and conditioning specific program is likely needed.

Despite the utility of these findings, it’s important to recognize certain limitations in the current study. Firstly, the relatively small sample size and the exclusive focus on elite youth female basketball players from a specific club may limit the generalizability of the findings to other player populations or different standards. To enhance the robustness of future research, larger and more diverse samples should be considered. Secondly, the utilization of a cross-sectional design in this study imposes limitations on the ability to establish causal relationships, given that measurements were captured at a singular time point (at the end of the season). Considering the potential influence of the season period on asymmetry values, which can be particularly important in the case of youth athletes [20], the implementation of longitudinal studies would offer valuable insights. Moreover, this study examined asymmetry in COD performance under controlled conditions. Real-game situations involve a myriad of factors, including opponents, court conditions, and psychological stressors, which may influence asymmetry differently. Therefore, future studies could investigate inter-limb asymmetry in competitive game settings to provide a more comprehensive understanding of its implications for performance and injury risk. Finally, future research should consider additional potential factors that might influence asymmetry and the COD performance, including hormonal fluctuations, prior injuries, training backgrounds, and movement mechanics.

## 5. Conclusions

In conclusion, this study highlights the presence of inter-limb asymmetry in COD performance among youth elite female basketball players reporting that the magnitude and direction of these asymmetries vary across different COD variables and tasks. Therefore, evaluations at different COD angles are needed. Moreover, the analysis of GRF holds the potential to provide a comprehensive profile of an athlete’s asymmetry, and COD and performance variables, which are “angle-dependent”. This multifaceted approach to assessment would yield a more holistic and informative depiction of inter-limb asymmetries and COD performance, which could have important implications for COD screening, injury reduction programs, and effective training interventions.

## Figures and Tables

**Table 1:** Mean test scores and test reliability data.

Test	Variables	Limb	Mean ± SD	ICC (95% CI)	CV (%)
COD 180°	Time (s)	Right	2.75 ± 0.15	0.91 (0.74–0.96)	5.41
		Left	2.74 ± 0.15	0.88 (0.71–0.92)	7.44
	GCT (s)	Right	0.73 ± 0.04	0.82 (0.66–0.90)	4.51
		Left	0.73 ± 0.03	0.82 (0.64–0.89)	3.72
	GRFx (anterior–posterior)	Right	258.99 ± 107.01	0.85 (0.62–0.91)	4.67
		Left	282.90 ± 83.30	0.84 (0.60–0.87)	5.54
	GRFy (mediolateral)	Right	46.38 ± 33.5	0.87 (0.73–0.92)	4.46
		Left	50.53 ± 28.45	0.86 (0.63–0.95)	5.21
	GRFz (vertical)	Right	841.48 ± 179.40	0.79 (0.46–0.92	6.01
		Left	898.76 ± 170.57	0.78 (0.41–0.92)	2.89
COD 70°	Time (s)	Right	2.31 ± 0.17	0.91 (0.78–0.97)	7.52
		Left	2.33 ± 0.13	0.94 (0.84–0.98)	6.41
	GCT (s)	Right	0.62 ± 0,02	0.94 (0.84–0.98)	1.32
		Left	0.61 ± 0.02	0.86 (0.62–0.95)	1.94
	GRFx (anterior–posterior)	Right	146.91 ± 52.93	0.91 (0.75–0.97)	2.85
		Left	139.59 ± 48.37	0.90 (0.74–0.96)	6.89
	GRFy (mediolateral)	Right	390.14 ± 61.79	0.89 (0.70–0.96)	1.61
		Left	401.51 ± 73.22	0.93 (0.81–0.97)	2.64
	GRFz (vertical)	Right	1386.44 ± 173.54	0.92 (0.80–0.97)	1.55
		Left	1349.60 ± 165.95	0.89 (0.71–0.96)	1.75

Key: COD = change direction capacity; GCT = ground contact time; GRF = ground reaction force; ICC = intraclass correlation coefficient; CI = confidence intervals; CV = coefficient of variation.

**Table 2:** Highest Performing versus Lowest Performing leg comparisons and asymmetry values for each variable in COD 180°.

	Time (s)	GCT (s)	GRFx (Anterior–Posterior)	GRFy (Mediolateral)	GRFz (Vertical)
HPL	2.72 ± 0.15	0.72 ± 0.03	308.59 ± 97.14	58.70 ± 31.19	932.67 ± 172.42
LPL	2.79 ± 0.14	0.75 ± 0.04	226.78 ± 72.84	46.57 ± 27.82	796.50 ± 152.64
*p* value	<0.001	0.003	<0.001	<0.001	<0.001
ES	1.78 (1.02–2.53)	0.82 (0.27–1.35)	1.18 (0.56–1.78)	0.98 (0.40–1.54)	1.24 (0.61–1.85)
ASI (%)	3.02 ± 1.73	3.96 ± 4.93	24.31 ± 17.39	20.9 ± 16.1	14.02 ± 10.41

Key: COD = change direction capacity; GCT = ground contact time; GRF = ground reaction force; ASI = asymmetry index; HPL = highest performing limb; LPL = lowest performing limb; ES = Cohen’s *d* effect size.

**Table 3:** Highest Performing versus Lowest Performing leg comparisons and asymmetry values for each variable in COD 70°.

	Time (s)	GCT (s)	GRFx (Anterior–Posterior)	GRFy (Mediolateral)	GRFz (Vertical)
HPL	2.27 ± 0.15	0.61 ± 0.01	164.66 ± 44.26	421.18 ± 66.11	1422.74 ± 149.24
LPL	2.39 ± 0.15	0.62 ± 0.02	129.77 ± 40.94	369.85 ± 56.87	1323.91 ± 170.17
*p* value	<0.001	<0.001	<0.001	<0.001	<0.001
ES	1.71 (0.96–2.43)	1.50 (0.81–2.17)	1.36 (0.71–2.01)	1.35 (0.69–1.98)	1.31 (0.65–1.92)
ASI (%)	5.09 ± 3.01	1.99 ± 1.51	21.7 ± 12.9	11.8 ± 7.79	7.07 ± 5.40

Key: COD = change direction capacity; GCT = ground contact time; GRF = ground reaction force; ASI = asymmetry index; HPL = highest performing limb; LPL = lowest performing limb; ES = Cohen’s *d* effect size.

**Table 4:** Kappa coefficients comparing asymmetry side consistency for each COD variable between COD 180° and COD 70° tests.

Test Comparison	Kappa Coefficient	Descriptor
COD time	0.143	Slight
GCT	0.087	Slight
GRFx (anterior–posterior)	0.024	Slight
GRFy (mediolateral)	−0.111	Poor
GRFz (vertical)	0.129	Slight

Key: COD = change direction capacity; GCT = ground contact time; GRF = ground reaction force.

## Data Availability

The data presented in this study are available on reasonable request from the corresponding author.
